# A Novel Biofilm Model System to Visualise Conjugal Transfer of Vancomycin Resistance by Environmental Enterococci

**DOI:** 10.3390/microorganisms9040789

**Published:** 2021-04-09

**Authors:** Michael Conwell, James S. G. Dooley, Patrick J. Naughton

**Affiliations:** Nutrition Innovation Centre for Food and Health [NICHE], School of Biomedical Science, Ulster University, Cromore Road, Coleraine Co., Londonderry BT52 1SA, UK; conwell-M1@ulster.ac.uk (M.C.); jsg.dooley@ulster.ac.uk (J.S.G.D.)

**Keywords:** enterococci, biofilm, model, antibiotic resistance, FISH

## Abstract

Enterococci and biofilm-associated infections are a growing problem worldwide, given the rise in antibiotic resistance in environmental and clinical settings. The increasing incidence of antibiotic resistance and its propagation potential within enterococcal biofilm is a concern. This requires a deeper understanding of how enterococcal biofilm develops, and how antibiotic resistance transfer takes place in these biofilms. Enterococcal biofilm assays, incorporating the study of antibiotic resistance transfer, require a system which can accommodate non-destructive, real-time experimentation. We adapted a Gene Frame^®^ combined with fluorescence microscopy as a novel non-destructive platform to study the conjugal transfer of vancomycin resistance in an established enterococcal biofilm.A multi-purpose fluorescent in situ hybridisation (FISH) probe, in a novel application, allowed the identification of low copy number mobile elements in the biofilm. Furthermore, a Hoechst stain and ENU 1470 FISH probe identified *Enterococcus faecium* transconjugants by excluding *Enterococcus faecalis* MF06036 donors. Biofilm created with a rifampicin resistant *E. faecalis* (MW01105^Rif^) recipient had a transfer efficiency of 2.01 × 10^−3^; double that of the biofilm primarily created by the donor (*E. faecalis* MF06036). Conjugation in the mixed enterococcal biofilm was triple the efficiency of donor biofilm. Double antibiotic treatment plus lysozyme combined with live/dead imaging provided fluorescent micrographs identifying *de novo* enterococcal vancomycin resistant transconjugants inside the biofilm. This is a model system for the further study of antibiotic resistance transfer events in enterococci. Biofilms promote the survival of enterococci and reduce the effectiveness of drug treatment in clinical settings, hence giving enterococci an advantage. Enterococci growing in biofilms exchange traits by means of horizontal gene transfer, but currently available models make study difficult. This work goes some way to providing a non-destructive, molecular imaging-based model system for the detection of antibiotic resistance gene transfer in enterococci.

## 1. Introduction

Biofilm-associated infections are a major cause of morbidity and mortality worldwide [1]. Treatment is complicated by difficulties with antibiotic delivery and recent evidence suggests that biofilm is a likely hotspot for antibiotic resistance transfer, thereby facilitating the development of multi-resistant strains [2]. Enterococci, including *Enterococcus faecalis* and *Enterococcus faecium*, are, after *Staphylococcus aureus,* the second most frequently isolated Gram-positive bacteria with associated antibiotic resistance [3] and, as with *S. aureus*, it is widely accepted that new ways to study and combat antibiotic resistance need to be developed [4]. Enterococci were previously associated mainly with urinary tract infections, but there is now a recognition of their importance in other infections [5,6], complicated by their association with organ transplants [7]. However, despite the number of models and approaches suggested [8,9], we have yet to develop reproducible means of identifying and visualising antibiotic resistance gene transfer in enterococci. Current models and the analysis of biofilms therein do not preserve the delicate structures of biofilm sufficiently for the study of horizontal gene transfer in biofilm [10].

Biofilm formation involves arrays of bacterial cells and extracellular polymeric substances adhered to a solid substrate [11,12]. Development of the biofilm both through cellular division and the introduction of new individuals occurs until a multispecies community has been established [13]. Biofilm models of development and biological function were initially based on *Pseudomonas aeruginosa* and *Pseudomonas fluorescens* research [14]. In terms of enterococci, models of biofilm development are not yet fully described, and an understanding of their biological function has only recently begun to emerge [8]. Enterococcal biofilm is an environment conducive for the exchange of information. This is facilitated by intercellular signalling pathways such as the *fsr* [15], pheromone-dependent and horizontal gene transfer (HGT) systems [16] and enterococcal surface proteins [17]. It may be that horizontal gene transfer within a biofilm is inefficient as compared to laboratory methodologies [18]. Indeed, Breuer et al. [19] demonstrated the likely disparate process and efficiency of enterococcal conjugation in the enteric environment as compared to their previous in vitro studies. There is high cell density and close contact amongst bacterial cells within a biofilm matrix. This, together with increased genetic competence and the accumulation of mobile genetic elements in the environment, creates a potential hotspot for the acquisition and spread of antibiotic resistance genes [20,21].

One of the major issues experienced in the identification of antibiotic resistance gene transfer in biofilm is the structural integrity of the biofilm itself. Our previous work has identified the importance of the pheromone-linked transfer of vancomycin resistance in enterococci [22] and in freshwater sponges [23]. Here, we have investigated how vancomycin resistance transfer can be identified and visualised in biofilm *in situ*. Many of the current techniques used to examine biofilm are destructive. They fail to protect the delicate underlying structures of developing biofilm and are unable to detect gene transfer. We have used a Gene Frame^®^ apparatus (GFA) in a novel application to provide support to a developing biofilm, allowing the development and maintenance of a delicate biofilm structure. We have combined this platform with the use of non-destructive fluorescence microscopy along with molecular techniques to detect vancomycin resistance gene transfer in enterococcal biofilm.

## 2. Materials and Methods

### 2.1. Bacterial Strains and Fluorescent In Situ Hybridisation (FISH) Probes

All *Enterococcus* isolates (Table 1) were retrieved from frozen stocks (−80 °C) and were maintained as previously described [22]. All probes were acquired through the ThermoFisher (Lutterworth, UK) custom DNA primers and, where specifically stated, Integrated DNA Technologies primer creation tools. The *van*A probes were conjugated with Fluorescein (FITC). The 16S *E. faecalis* and 23S *E. faecium* rRNA FISH probes first used by Wellinghausen et al. [24] were used here to identify previously characterised *E. faecalis* and *E. faecium* (Table 1) [22]. These probes were 5′ conjugated with Alexa fluor 594 and used to confirm all enterococci in biofilms. The multiplexing PCR technique has previously been described for simultaneous enterococcal vancomycin resistance gene detection [25,26]. Using the same principles to select a suitable primer set for multiple PCR, we selected two probe sequence targets on the enterocococcal *van*A (NCBI Reference SequenceNC_014475.1) cluster which would be compatible in a multiplexing reaction. We used primer BLAST against all deposited *van*A gene sequences in the NCBI database to ensure specificity. These probes were crosschecked for stability using the PCR primer statistics tool in the sequence manipulation suite [27]. Analogous to the detection of low content number targets first highlighted by Pernthaler et al. [28], we used two oligonucleotide probes simultaneously. These were utilised in a hybridisation reaction targeted to the same gene to produce a compounding increase in the fluorescence signal. All reactions were controlled using *E. faecalis* ATCC 29212 with *S. aureus* ATTC 43300 employed as a non-specific control for *Enterococcus* probes. All chemicals and antibiotics used were sourced from Sigma-Aldrich (Dorset, UK), unless otherwise stated.

### 2.2. Culture Conditions for Growth of Enterococcal Biofilm

All enterococcal biofilms were grown in Tryptone Soya Broth (TSB), (CMO129, Oxoid, UK), (1% glucose) for 24–48 h statically at 37 °C. Briefly, enterococci were grown in TSB to approximately 2.5 × 10^9^ CFU/mL. Cells were harvested, resuspended and diluted 1:200 in TSB (1% glucose) and inoculated onto all surface conditions (below). For conjugation reactions in the GFA, cellular inoculations were approximately 2.5 × 10^6^ CFU/mL. Prior to inoculation all surfaces were coated with gelatin (Sigma-Aldrich, Dorset, UK). Gelatin was prepared as a 2% (*w*/*v*) stock in tissue grade H_2_O (ThermoFisher, Lutterworth, UK), autoclaved and coated on substrate (10 μL/cm^2^) and dried in a tissue culture hood for 2 h. For coverslip biofilms, 16-well polystyrene microplates (ThermoFisher, Lutterworth, UK) with a 10 mm circular glass coverslip (ThermoFisher, Lutterworth, UK) were coated in gelatin and placed flat along the bottom of each well and used to grow enterococci. For microplate biofilms, enterococci were grown in 48-well polystyrene microplates (ThermoFisher, Lutterworth, UK) coated with gelatin. For GFA biofilms, double-sided adhesive Gene Frames^®^ were attached to glass microscope slides (ThermoFisher, Lutterworth, UK) coated with gelatin. These were UVC sterilised for 45 min according to manufacturers’ instructions (CL-100 UV Crosslink, UVP, UK). Enterococcal biofilm inocula were prepared and placed within the Gene Frame well inside a tissue culture hood and sealed using the top side adhesive and supplied coverslips.

### 2.3. Characterisation of the GFA Grown Biofilm

Concanavalin A (Con-A) Texas red conjugate (Invitrogen, Renfrew, UK) was used to stain the enterococcal biofilm. A stock solution of 1 mg/mL in 0.1 M sodium bicarbonate (pH 8.3) (with 2 mM sodium azide added before storage) was diluted to working concentrations of 25–50 µg/mL with incubation times of 30–60 min at room temperature (RT).

### 2.4. Quantification of Cell Biomass

Quantification was carried out on raw image files for cellular staining (Hoechst) and extracellular polymeric substance (EPS), (Con-A) converting staining to 8 bit images using ImageJ (NIH). Data was analysed using GraphPad Prism (Version 6.0).

### 2.5. Fluorescent Imaging of Enterococci in Biofilm

Biofilm staining was performed in situ using final staining volumes of 100 µL. Biofilm was washed three times with 1000 µL of phosphate buffered saline (PBS). Hoechst 33342 (Sigma-Aldrich, Dorset, UK) DNA stain stocks of 10 µg/mL in DMSO were optimised for detecting enterococci at a working concentration (diluted in PBS) 2.5 µg/mL for biofilm. All Hoechst incubation times were 15 min at RT.

The live/dead *Bac*Light bacterial viability kit L7012 which included the SYTO9 stain (ThermoFisher, Lutterworth, UK) was used for live/dead staining of enterococci as per manufacturer’s instructions. Stains were applied at a concentration of 0.003% *v*/*v* and incubated at RT for 20 min for enterococcal biofilms. Stains were washed with PBS (×3, dried and mounted using mounting medium (Vectashield) for fluorescence microscopy. Microscopy was carried out with a 100× objective on a Nikon eclipse E400 with a Nikon DS-fi1c using a G2-A and UV filter set. Images were captured with NIS-elements and ImageJ (NIH).

### 2.6. Detection of Conjugation in Biofilm using the Gene Frame^®^

**Figure 1:**
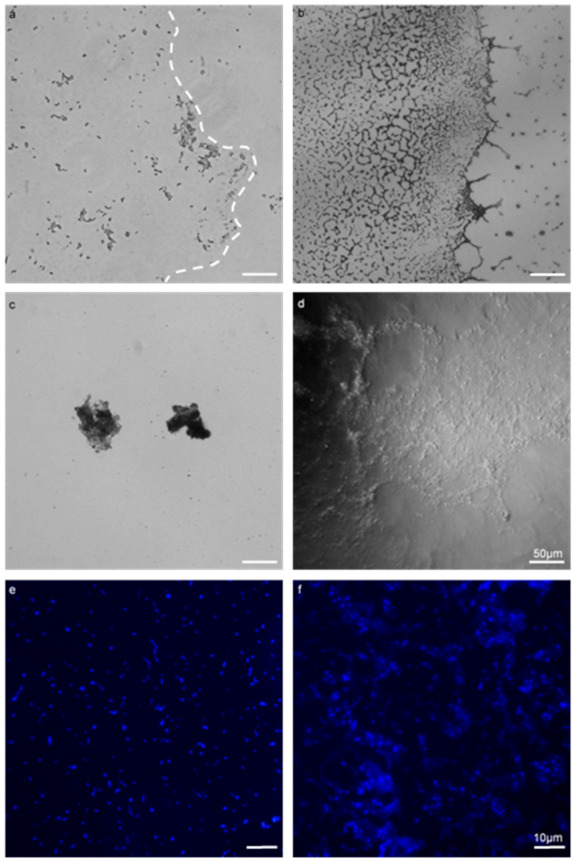
Comparison of a biofilm assay on a coverslip with the Gene Frame^®^ apparatus (GFA) applied to *E. faecalis* MF04010. All biofilms were grown for 24 h at 37 °C under static growth conditions. (**a**,**b**) 40× micrographs of the coverslip method (**a**) and the Gene Frame (**b**). (**c**,**d**) 40× phase contrast micrographs (**c**), highlighting free-floating, detached biofilm masses that occurred when processed for microscopic visualisation (**d**). The Gene Frame method generated minimal biofilm surface detachment. (**e**,**f**) 100× micrographs imaged with fluorescence and Hoechst DNA staining (blue) (panels **e**,**f**). The coverslip method (**e**) dislodged large quantities of cells into the planktonic phase through processing. The gene frame (**f**) minimised this phenomenon. Scale bar for images (**a**–**d**) represents 50 microns. Scale bar for (**e**,**f**) represents ten microns.

Gene Frames^®^ (ThermoFisher) are typically used for in situ hybridisation and PCR reactions to detect genetic targets in histological tissue sections [30,31]. Gene Frames are superior to hydrophobic pens and parafilm^®^ as they are more robust for multistep experimental approaches. They are also less damaging to materials under study and prevent the evaporation of testing solutions [32]. The Gene Frame is used in the current study and developed as the Gene Frame Apparatus (GFA), providing a model system to study conjugation in biofilm. It provides a novel approach to develop a scaffold for analysing biofilm development coupled with gene detection in a non-destructive manner. Biofilm development in the GFA was initially examined using phase contrast microscopy (see Figure 1), [22]. A biofilm-forming *van*A donor strain (MF06036) was added (30 µL of TSB 1% glucose culture at 2.5 × 10^6^ CFU/mL) to a Gene Frame on a gelatin-coated slide (10 µg/cm^2^), sealed and incubated at 37 °C for 24 h. The plastic seal was aseptically removed, and the biofilm washed in sterile PBS to remove planktonic cells. The recipient strain (MW01105^Rif^) was added (30 µL of TSB 1% glucose culture at 2.5 × 10^6^ CFU/mL) to the pre-established biofilm, sealed and incubated for a further 24 h at 37 °C. The Gene Frame was removed, and biofilm was washed with PBS. The biofilm was then scraped off and homogenised in dimethyl sulfoxide (DMSO) (30 µL) to prevent aggregation [18]. The homogenate was added to double selection plates (vancomycin 10 µg/mL and rifampicin 100 µg/mL) and incubated for 24 h at 37 °C. Resultant transconjugant colonies were enumerated as described by Conwell et al. [22]. Transfer efficiencies were calculated as number of transconjugants per donor.

Variations on the conjugation of enterococci within biofilm were performed as follows. A “donor” biofilm was one whereby the donor (MF06036) was used to initiate biofilm formation and the recipient (MW01105^Rif^) was added to the pre-established biofilm. A “recipient” biofilm was one whereby the recipient (MW01105^Rif^) was used to initiate biofilm and the donor (MF06036) was added to the pre-established biofilm. A “mixed” biofilm was one whereby both partners were added simultaneously to initiate biofilm formation.

### 2.7. Fluorescent In Situ Hybridisation (FISH) Detection of Enterococcal Cells and vanA Antibiotic Resistance Gene in Biofilm

Hybridisation procedures to detect enterococcal cells were adapted from Waar et al. [33]. Enterococcal biofilms were first grown in the GFA for 48 h at 37 °C under static growth conditions. Coverslips were carefully removed, leaving the Gene Frame intact surrounding the biofilm. Biofilms were then washed with PBS and fixed in 97% ethanol for five minutes. The GFA incorporating the fixed biofilms were treated (in a humidified hybridisation chamber) with lysozyme (1 mg/mL) for 30 min at 37 °C. After 30 min, the biofilm was probed (Table 2) with either ENF 191, or ENU 1470 (10 ng/µL) in 10% formamide for 2–24 h at 50 °C. Cells were washed (3 × 5 min) in wash solution (0.9 M NaCl, 20 Mm Tris-HCl (pH 7.5) at RT and air dried. The GFA was removed from the humidified chamber and the slides were mounted with Vectashield^®^ mounting medium for fluorescence microscopy. Slides were imaged with a 100× objective on a Nikon eclipse E400 with a Nikon DS-fi1c using a G2-A and UV filter set. Images were captured with NIS-elements and ImageJ (NIH).

For fluorescent in situ hybridisation of vancomycin-resistant enterococci containing the *van*A gene, biofilms were prepared as above with the following modification. The vancomycin-resistant *E. faecalis* MF06036 was grown for 48 h using biofilm inoculum and growth media that contained 10 µg/mL vancomycin. Pre-treatment with vancomycin was essential for a strong signal. The standard FISH assay described by Warr et al. [33] was modified to target two binding sites across the *van*A vancomycin resistance gene (NC_014475.1). The two probes selected that bound *van*A were designed to be the same length and have the same annealing temperature. This design approach eliminated multiple annealing steps within the protocol. Fixed biofilms were incubated with the two distinct fluorescein-labelled FISH probes specific to *vanA* (Table 2) for 2–24 h at 50 °C in 10% formamide at 50ng/µL. An Alexa fluor 594 probe targeted to *E. faecalis* 16S rRNA (ENF 191) was used as a control. Slides were imaged as described above.

### 2.8. Visualisation of Enterococcal Transconjugants in a Conjugal Biofilm

Enterococcal conjugations in biofilm using the Gene Frame^®^ were carried out as above between MF01105^rif^ and MF06036. Post-conjugation, antibiotic selection (10 µg/mL vancomycin and 100 µg/mL rifampicin, or tetracycline 30 μg/mL) was applied directly to the conjugal biofilms incubated at 37 °C for 24 h. Lysozyme (2 mg/mL) was then added for an additional 24 h at 37 °C. Biofilms were then subjected to live/dead and FISH staining protocols and imaged with a 100× objective on a Nikon eclipse E400 with a Nikon DS-fi1c using a G2-A and UV filter set. Images were captured with NIS-elements and ImageJ (NIH).

### 2.9. Statistical Analysis

Assays performed on the GFA were performed five times with six biological repeats. Microscopy-based statistics came from 10 regions of interest with three independent repeats. Averages were taken and when appropriate standard error of the mean was displayed. Significance was computed using GraphPad Prism 6 t-test function, one way and two-way analysis of variance.

## 3. Results and Discussion

### 3.1. Growth of Biofilm in GFA

The use of the GFA permitted the growth and visualisation of enterococcal biofilm by employing as little as possible physical processing and manipulations to samples (Figure 1). This was found to be superior to the coverslip and polystyrene microplate method. The coverslip method required manipulation of the slips with forceps, with inversion and mounting to a slide for microscopic visualisation. These processes could be omitted when using the GFA. The microplate method required significant micropipette aspirating when staining, whereas the GFA could be stained with decanting and tissue aspiration. From Figure 1, the coverslip method (a) was employed against the Gene Frame (b). The coverslip method retained a small quantity of the biofilm post-processing, evident by the lack of extracellular content within the biofilm boundary (dashed line). The Gene Frame method produced biofilm with a high density that retained the majority of extracellular content within the biofilm boundary.

Figure 1c highlights free-floating, detached biofilm masses that occur when processed for microscopic visualisation from the coverslip method; whereas in Figure 1d the Gene Frame method generated minimal biofilm surface detachment when processed for microscopic visualisation. There were zero instances of free-floating biofilm mass in the Gene Frame experiments. Cell retention differences between polystyrene microplate (e) and Gene Frame (f) methods were apparent. The microplate method rends large quantities of cells into the planktonic phase; whereas the Gene Frame sufficiently minimised this phenomenon, but not without total elimination.

### 3.2. Characterisation of Biofilm Using Con-A Staining of EPS

**Figure 2:**
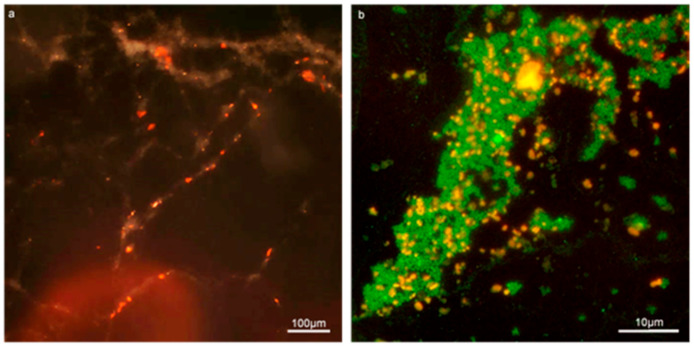
Con-A and SYTO9 effectively stains the EPS and cellular content of enterococcal biofilm in the Gene Frame biofilm apparatus (GFA). (**a**) 10× composite micrograph of 24-h biofilm produced by MF04010 using a dark field and the G2-A filter to capture the red fluorescence staining of Con-A bound to the EPS. (**b**) 100× fluorescent micrograph of MF06036 24-h biofilm stained for cellular content with SYTO9 (green) and EPS with Con-A (red).

**Figure 3:**
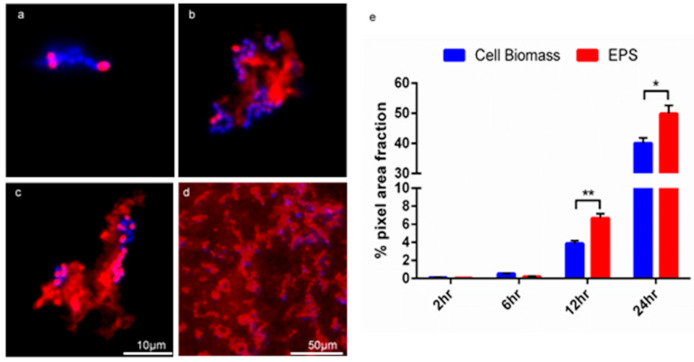
The GFA can be used to visualise and quantitate the biofilm development of MF04010. Biofilms were stained with Con-A (red) and Hoechst (blue) and imaged with florescence microscopy. (**a**) 100× micrograph of 2-h biofilm growth. (**b**) 100× micrograph of 6-h biofilm growth. (**c**) 100× micrograph of a 12-h biofilm growth. (**d**) 40× micrograph of a 24-h biofilm growth. Scale bar for 100× micrographs (**a**–**c**) represents 10 microns. (**e**) Graph of quantification of cell biomass staining versus EPS staining of MF04010. The Hoechst and Con-A micrographs from the biofilm growth of MF04010 were imported into ImageJ. Micrographs were converted to 8-bit images and the % grey pixel areas were computed as per the ImageJ analyse-measure function. At the 12-h time-point the *p* value between cell biomass and EPS was * 0.0001. At the 24-h time-point the *p* value between cell biomass and EPS was ** 0.0083.

The Con-A staining of the enterococcal biofilm matrix is illustrated in Figure 2 and Figure 3. Figure 2 shows clustered staining under low magnification as well as punctuated staining of inclusions, shown under higher magnification in Figure 3. Early set down and formation of MF04010 biofilm was observed using the GFA, as seen in Figure 3a–d. Figure 3a was imaged 2 h after cells were added to the GFA. The attachment sites and production of EPS can be observed with the Con-A staining (red). At 6 h, Figure 3b demonstrates large quantities of extracellular material stained with Con-A. At 12 h, Figure 3c shows greater proportions of EPS (red) compared to the cellular biomass (blue overlapping red) alone. Figure 2e shows significant quantification of EPS at 12 h (*p* = 0.0001) and 24 h (*p* = 0.0083).

### 3.3. Detection of vanA in Biofilm Using Multi-Probe FISH

**Figure 4:**
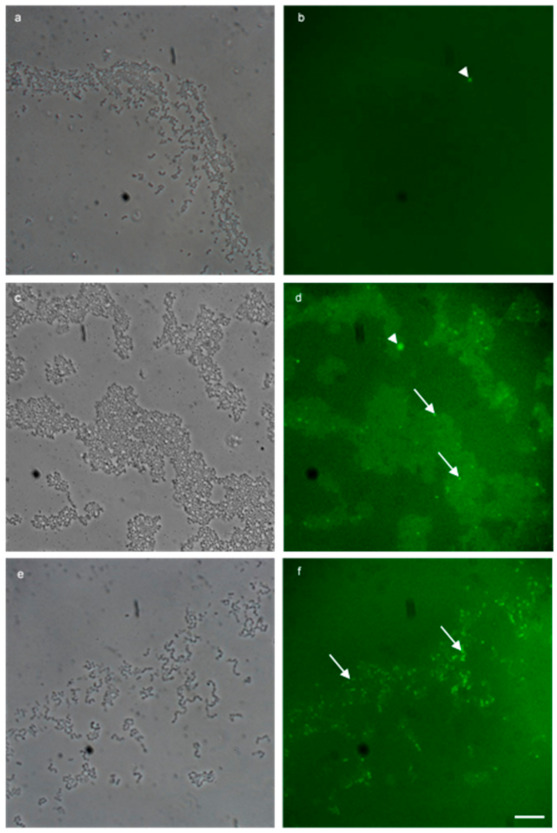
Multi-probe FISH successfully detected plasmid bound *van*A fluorescein (green) resistance genes in enterococcal biofilm. (**a**,**b**) 100× micrograph overlays of a negative biological control using MF04010 and fluorescein probes targeted to the *vanA* vancomycin resistance gene. (**a**) Phase contrast imaging to highlight MF04010, 48-h biofilm cells grown with TSB (1% glucose) at 37 °C incubated statically. Media was replaced at the 24-h time point. (**b**) Green fluorescent imaging of the same region. (**c**,**d**) 100× micrograph overlays of the addition of sub inhibitory (10 µg/mL) vancomycin for 34 h, to the *vanA* positive MF06036 in a 24-h pre-established biofilm grown with TSB (1% glucose) at 37 °C. (**c**) Phase contrast imaging MF06036 vancomycin exposed biofilm. (**d**) Green fluorescent imaging of the same region. (**e**,**f**) 100× micrograph overlays of the addition of sub inhibitory (10 µg/mL) vancomycin to the biofilm formation media TSB (1% glucose) during the formation of isolate MF06036′s biofilm (24 h). Media was replaced at the 24-h time point with vancomycin TSB (1% glucose) for an additional 24 h. (**e**) Phase contrast imaging MF06036 vancomycin exposed biofilm. (**f**) Green fluorescent imaging of the same region. Scale bar represents ten microns. Arrowhead—fluorescence artefact, arrow—cell positive for *vanA* staining.

FISH detection of plasmid-bound *van*A is shown in Figure 4 by the application of the *van*A probes. Figure 4 a, b examines the *van*A negative staining control. Figure 4a shows phase contrast imaging of MF04010 biofilm grown (48 h) in TSB with 1% glucose under static conditions. Figure 4b shows green fluorescence imaging of the same region. Figure 4c,d show the addition of (10 µg/mL) vancomycin for a further 24 h, to the *van*A positive MF06036 in a 24-h pre-established biofilm grown with TSB (1% glucose) at 37 °C. Figure 4c shows phase contrast imaging of MF06036 vancomycin exposed biofilm along with green fluorescence imaging of the same region, shown in Figure 4d. Figure 4e,f show a 100× micrograph of the addition of (10 µg/mL) vancomycin to the biofilm formation media TSB (1% glucose) during the formation of isolate MF06036′s biofilm (24 h). Media was replaced at the 24-h time point with (10 µg/mL) vancomycin TSB (1% glucose) for an additional 24 h, and Figure 4e shows the phase contrast imaging of MF06036 vancomycin exposed biofilm. Figure 4f shows green fluorescent imaging of the same region.

### 3.4. Detection of vanA Transconjugants in Biofilm

**Figure 5:**
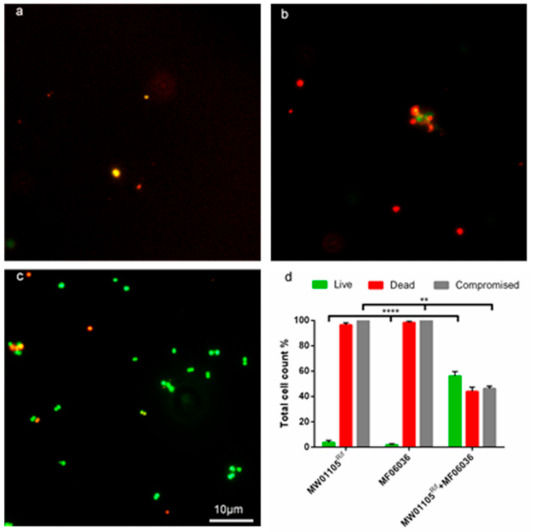
Application of double antibiotic selection and lysozyme degradation permitted the successful selection of transconjugants inside 24-h biofilms. (**a**) 100× micrograph of a biofilm made with MW01105^Rif^. (**b**) 100× micrograph of a biofilm made with MF06036. (**c**) 100× micrograph of a conjugation reaction with MW01105^Rif^ and MF06036 inside biofilm. Subfigures (**a**–**c**) were visualised with live/dead BacLight staining (green/red) after treatment with double selection (10 µg/mL vancomycin, 100 µg/mL rifampicin) to inhibit/kill cells. The biofilm was then treated with lysozyme (2 mg/mL) for a further 24 h to eradicate the compromised cells. (**d**) A graph showing the total cell count of the three biofilms visualised in (**a**–**c**). Error bars represent the standard error of the mean of each cell count group for MW01105^Rif^, MF06036 and MW01105^Rif^ + MF06036. Experiments were carried out with six biological repeats and five independent repeats. Statistics generated from each repeat used 10 regions of interest. Scale bar represents ten microns. Significance **** *p* = 0.0001, significance ** *p =* 0.002.

**Figure 6:**
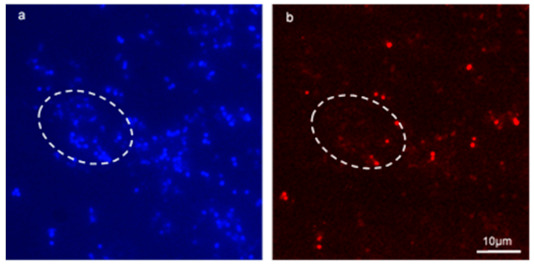
Application of FISH on a mixed-species enterococcal conjugation reaction inside biofilm successfully identified transconjugants. (**a**,**b**) are fluorescent micrographs of a conjugation reaction between *E. faecalis* MF06036 and *E. faecium* ST01109^Rif^ inside biofilm. Biofilm was created, and enterococci allowed to conjugate for 24 h. Post-conjugation, biofilm was treated with tetracycline (30 μg/mL) for 24 h, and then treated with lysozyme (2 mg/mL) for an additional 24 h. (**a**) 100× fluorescent micrograph showing Hoechst staining of tetracycline and lysozyme treated biofilm post-enterococcal conjugation. (**b**) FISH staining of same region as (**a**) with probes specific for *E. faecium* only. White dashed ovoid depicts region of interest with total cell count stained blue (**a**) and stained red (**b**) with *E. faecium* cells only.

A biofilm conjugation reaction was established with the recipient (either MW01105^Rif^ (Figure 5) or ST01109^rif^ (Figure 6) and donor MF06036. Isolates MW01105^rif^ and MF06036 were used previously to demonstrate the transfer of vancomycin resistance by conjugation [22]. Figure 5 quantifies the successful isolation of the *van*A transconjugants from conjugation reactions between the recipient and donor in three different biofilm reactions, as described in the methods. Biofilm created with MW01105^Rif^ had a transfer efficiency of 2.01 × 10^−3^ (150.75 CFU); double that of the biofilm primarily created by the donor (biofilm created with MF06036) at 1.01 × 10^−3^ (75.75 CFU). Conjugation in the mixed enterococcal biofilm was three times as efficient as the donor biofilm with an efficiency of 3.04 × 10^−3^ (228 CFU). Statistically there was no difference between these three types of biofilm conjugation tests, excluding a comparison of the donor-only biofilm against the mixed biofilm (*p* value of 0.0048 ** using Welch’s correction). However, a mixed biofilm (without accounting for planktonic enterococci) yielded an improvement in the generation of transconjugants.

The GFA has the disadvantage that visualisation of transconjugants can be difficult in biofilms of high cell density. Therefore, we set out to eliminate the parent strains in the biofilm so that visualisation of the transconjugants would be possible. Figure 5a–c clearly demonstrates microscopically the synergistic killing of MW01105^Rif^ (a), MF06036 (b) and the successful conjugation of MW01105^Rif^ and MF06036 (c) highlighted with live SYTO9 green imaging. Figure 5d is a total cell count of the three biofilms visualised in (a–c). As this was a biofilm conjugation experiment with MW01105^Rif^, MF06036 and freshly created transconjugants, these increased numbers of dead and structurally compromised cells were expected.

### 3.5. Visualising HGT between Species

Figure 6a, bare fluorescent micrographs of a mixed-species biofilm conjugation reaction between *E. faecalis* MF06036 and *E. faecium* ST01109^Rif^. Due to the selection conditions, the only remaining enterococci in this biofilm were MF06036 (all blue stained cells that do not co-localise with red staining) and the resultant transconjugants from the conjugation reaction, which was stained with the FISH probe. Washing steps during the methodology ensured planktonic cells were washed away and binding controls ensured that non-specific signals did not interfere with data collection. The Hoechst photo-conversion did not enter the B2-A blue filter on the fluorescent microscope. This assay provided visual evidence of enterococcal conjugation within biofilm, by way of specifically labelling surviving recipients from donors within undisturbed biofilm. This approach permitted the visualisation of transconjugants ensured to be only created from within biofilm.

### 3.6. Application of Gene Frame Apparatus Model to Biofilm Studies

In our hands, the GFA model has been shown to have several advantages over traditional visualisation methods [34], principally minimal surface detachment and increased cellular density. The GFA allowed the imaging of the early stages of biofilm formation (Figure 2 and Figure 3) using a standard fluorescence microscope in a non-destructive manner. Figure 2a depicts the diffuse nature of MF04010 biofilm, an observation not apparent in every isolate, for example MF06036 (Figure 2b). Biofilm from MF06036 formed with an apparent high cellular density of individual coccoid cells (Figure 2b). This morphology is atypical of MF06036 as it normally appears in diplococcus form in planktonic phase [22]. Biofilm formation capability is a function of cell attachment to a solid substrate, adhesion and growth [8,35,36,37]. There are also distinctive biofilm formation variations based on static or laminar flow growth conditions [38]. Most assays manipulate some of these characteristics to optimise biofilm biomass and produce an easily measurable signal. However, this approach may have a negative impact on the functionality of bacteria in the biofilm state, such as a model of in vivo persistent antibiotic-resistant infection [39]. There is a growing consensus that bacteria modulate their biofilm to adapt to changing conditions of stress, rather than simply producing biofilm in large quantities irrespective of external conditions [38,40,41].

The novel application of the GFA here to biofilm studies allowed the visualisation of what has been identified as fragile enterococcal biofilm [42]. The majority of current techniques, including the polystyrene microplate assay [43] and submerged coverslip biofilm formation assay [44], can be destructive to the delicate structures of biofilm, thus making in-depth analysis of interactions including visualisation of antibiotic resistance genes extremely difficult [29]. In our hands, the GFA has been shown to have several advantages over traditional visualisation methods [45], principally minimal surface detachment and increased cellular density (Figure 1). This unique characteristic of the GFA allowed unprocessed, undisturbed imaging of 24–48-h biofilm (Figure 1) using a standard fluorescence microscope in a non-destructive manner.

### 3.7. Deployment of Con-A to Identify EPS in Biofilm

The lectin, Con-A, binds to α-mannopyranosyl and α-glucopyranosyl residues of carbohydrates [46]. Enterococcal cell wall components feature lipoteichoic acids with kojibiose containing an α-D glucopyranosyl residue [47]. Con-A binds to polysaccharide residues and so, attached to a suitable fluorochrome, it can be used as a selective stain to examine biofilm formation and EPS production [48,49]. Previously, Con-A has been used to identify various types of cells, including those found in archaeal and bacterial biofilm [50]. Hence, the capacity of Con-A to bind to a range of capsular/cell-associated structures suggests similarities in EPS composition. It has been used previously in our laboratory to show reduced EPS production in a *Burkholderia thailandensis* transposon mutant [51]. Fluorescently labelled Con-A was also shown to stain the α-linked mannose and α-linked D-glucose components of enterococcal EPS [52]. In the current study, the Con-A Alexa Texas red staining of EPS eclipses DNA staining due to continual EPS formation in the biofilm (Figure 3). In comparison with the diplococcus form, the lack of staining where enterococci form chains, suggests lower EPS/biofilm formation.

### 3.8. FISH Visualisation of vanA Transconjugants

The GFA allowed for the development of a multi-probe FISH protocol for easy analysis of enterococci in biofilms, and for the first time, allowed the visualisation of *vanA*-containing cells. In the current study, probes from a commercial supplier were all the same length and had the same annealing temperatures much like a multiplex PCR [53]. FISH assays have been previously used to monitor the presence of enterococci in faecal matter and activated sludge from wastewater treatment plants using genomic identifiers such as 16S or 23S rRNA [54,55]. In the current study, the novel application of FISH identified low copy number AMR mobile genes without molecular amplification steps. Previous modifications to the FISH protocol have included introducing reporter genes into plasmids and replicating the modified plasmid into *Enterococcus* [18]. The multi-probe oligonucleotide FISH developed here, to target a single enterococcal gene in biofilm state, follows Zwirglmaier et al. [56] and their multi-fluorophore FISH probe designed to target single genes. The current study reduces some of the complexity and cellular internalisation limitations imposed by the RING FISH probes [56]. Crucially, selective pressure of vancomycin (10 µg/mL) during the formation of the MF06036 biofilm, in tandem with the increased *van*A double probe concentrations (50ng/µL) and incubation, is essential to secure a signal. The signal is lost completely when standard probe concentrations and incubation time are used with only a single *van*A probe. Therefore, the improvements to the protocol highlighted here were crucial in the identification of *vanA* within *E. faecalis* MF06036 without the need for complex amplification steps. This type of FISH reaction has also been used to quantitate the physical transcripts involved in the conjugation of the pCF10 plasmid in *E. faecalis* [19].

The GFA additionally allowed for a multi-stage *vanA* conjugation assay to be demonstrated in enterococci in the biofilm state, whilst eliminating planktonic cells from any results. The nature of this protocol (Figure S1) ensured that planktonic conjugation was eliminated due to the removal of all non-adherent cells prior to the addition of the conjugation partner.

### 3.9. In Situ Detection of Transconjugants

The facilitation of conjugation reactions to occur inside biofilm, then applying both the selective pressure of antibiotics (rifampicin and vancomycin) and the action of lysozyme, identified transconjugants situated within enterococcal biofilm for the first time (Figure 5). The *vanA* transconjugants had combined vancomycin, rifampicin and lysozyme MICs four times higher than the donor and eight times higher than the recipient (data not shown). This resulted in the selective killing and lysozymic degradation of the parent strains, making microscopic visualisation of the surviving transconjugants possible. An additional experiment was performed, whereby an interspecies conjugation reaction was examined using FISH and double selection (tetracycline and lysozyme), highlighting only the transconjugants from *E. faecium* ST01109^Rif^ (Figure 6a,b). Any remaining *E. faecium* ST01109^Rif^ recipients were eliminated by the double selection, thus excluding any non-specific live/dead staining of the transconjugant. Furthermore, the Hoechst stain and the ENU 1470 FISH probe selected for *E. faecium* transconjugants (excluding *E. faecalis* MF06036 donors). This double antibiotic treatment plus lysozyme combined with live/dead imaging provided the first report of fluorescent micrographs identifying *de novo* transconjugants inside biofilm.

## 4. Conclusions

Biofilm studies are assay-dependent, whether it is a measure of biofilm formation based on cell counting alone, the ratio of cells to biomass or dry biomass alone [57,58,59,60,61]. There is a clear need for a consensus regarding the standardisation of growth conditions for enterococci prior to carrying out detailed experimental studies. There is also a need to refine the methods available for the detection of resistance genes so they can be applied to biofilms/biofilm studies. The multi-probe FISH developed in the current study is a simple, cheap and novel method for the examination of enterococcal biofilms from environmental isolates containing mobile resistance, e.g., *van*A genes, using epifluorescence. However, it is well understood that enterococci form biofilm in many environments, e.g., river basins, medical devices, animal intestine and circulatory systems [62,63,64,65]. These environments all have, to varying degrees, conditions of laminar and turbulent flow, which the GFA cannot adequately replicate *in vitro*, as it has been designed to facilitate enterococcal biofilm processes under static conditions. Indeed, several enterococcal pathologies involve translocation from catheter and intestinal colonisations to bacteraemia and endocarditis [36,66]. Antibiotic-resistant enterococcal infection has been implicated from river ecosystems with proximity to farming processes and urban wastewater effluent [67,68,69]. Therefore, devices such as the BioFlux flow-through device and drip flow biofilm reactor, which are designed to grow biofilms under flow, should be considered against the GFA [70,71]. The BioFlux flow-through device is a bespoke microfluidic device that uses etched microtiter plates to grow biofilm under fluidic flow, which is highly controllable. However, it cannot be used to study cellular processes within biofilm in great detail. It cannot be opened and have other constituents added or removed in compounding multistage experiments, as compared to the GFA which has this capability. The drip flow biofilm reactor forms biofilms across a coupon or microscope slide, titled at an angle whereby media runs across the substrate, inducing flow. However, the fact that the coupon needs to be removed for processing, and biofilms cannot be imaged *in situ*, significantly reduces the overall functionality of this device as compared to the proposed GFA. We suggest that in future studies the GFA could be modified to introduce a microfluidic chamber to capture biofilm modalities from fluidic environments whilst still maintaining the unique advantages offered with the current setup of this device. The GFA has the potential to allow the study of any bacterial biofilm without the limitations applied from the other techniques discussed in this study. Pathogens with similar motility characteristics, such as *S. aureus*, could be visually studied in tandem with the enterococcal *van*A transfer mechanism within the GFA as an example. This potential is compounded when regarding “poor” biofilm-forming isolates as defined by the traditional techniques, or even isolates which may require specific substrate compositions such as gelatin coating [72,73]. Substrate modifications can be applied directly within the GFA, or the frame itself can be applied to laboratory-specific substrates for study. Indeed, for enterococci alone there are a wide range of techniques and growth media utilised, thus, raising questions into their effectiveness and trueness relative to natural biofilm formation [42]. It is a cheap, commercially available apparatus that has the capability of being used for real-time and end point biofilm tracking. Since it can be applied to any substrate, this allows for universal compatibility with any microscope or imaging system, not just expensive bespoke packages.

Improved model systems, including the GFA proposed here, solves the complex problem of how to visualise biofilms whilst maintaining important structural characteristics. These systems could give us a better understanding of the drivers of horizontal gene transfer in biofilm, and methods to study them could contribute to efforts to reduce the spread of resistance genes and prolong the useful life of antibiotics.

## Figures and Tables

**Table 1:** Bacterial strain information.

Isolate	Species	Resistance	Biofilm Production	Conjugation Role	Reference
MF06036	*E. faecalis*	Van, Ery, Tet	Moderate	Donor	[22]
MW01105^Rif^	*E. faecalis*	Rif	Moderate	Recipient	[22]
ST01109^Rif^	*E. faecium*	Rif	Moderate	Recipient	[22]
MF04010	*E. faecalis*	Tet, Gen	Moderate	Donor	[29]

Van—vancomycin, Ery—erythromycin, Tet—tetracycline, Rif—rifampicin, Gen—gentamycin.

**Table 2:** Probes used for fluorescent in situ hybridisation (FISH).

Target	Probe	Sequence 5′ to 3′	Reference
*Enterococcus vanA*	*vanA* 1	GCAAGTCAGGTGAAGATGGA	This study
	*vanA* 2	AGGAGCATGACGTATCGGTA	This study
*E. faecalis* 16S rRNA	ENF 191	GAAAGCGCCTTTCACTCTTATGC	[24]
*E. faecium* 23S rRNA	ENU 1470	GACTCCTTCAGACTTACTGCTTGG	[24]

## Data Availability

The data presented in this study are available on request from the corresponding author.

## Supplementary Materials

The following are available online at https://www.mdpi.com/article/10.3390/microorganisms9040789/s1, Figure S1: Transfer efficiencies of the donors (MF06036 and MW01105^Rif^) and the mixed biofilm (MW01105^Rif^+ MF06036) expressed as CFU±SEM; Figure S2: The gene frame apparatus (GFA): A novel biofilm development tool for microscopic visualisation of fragile enterococcal biofilm.
