# Supplementary material for: A Novel Biofilm Model System to Visualise Conjugal Transfer of Vancomycin Resistance by Environmental Enterococci
Source: Microorganisms. 2021 Apr 9;9(4):789. doi: 10.3390/microorganisms9040789 (PMC8070047; doi:10.3390/microorganisms9040789)
Supplement: Supplementary file 1 [file microorganisms-09-00789-s001.zip › Supplementary Figure S1.pdf]

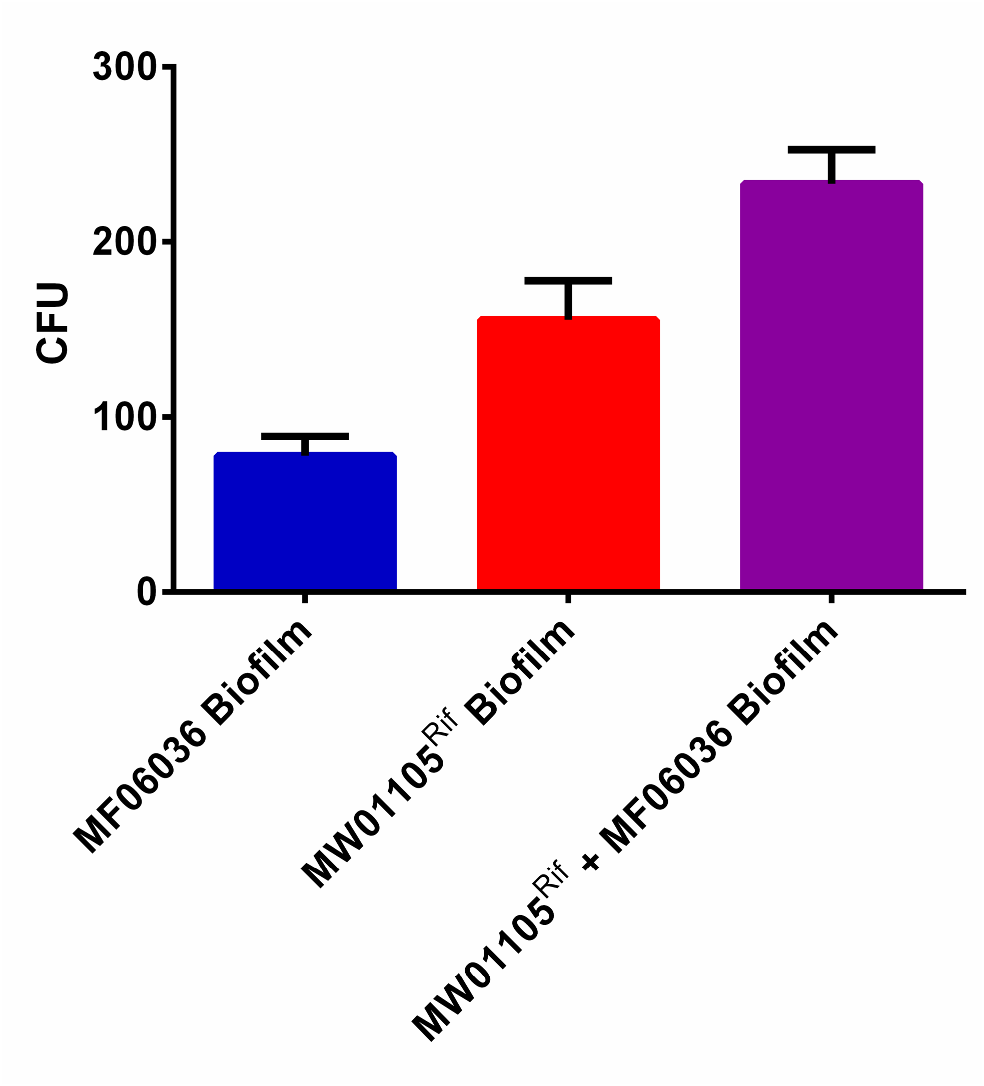

Supplementary Figure S1. Transfer efficiencies

Transfer efficiencies of the donors (MF06036 and MW01105^{Rif}) and the mixed biofilm (MW01105^{Rif}+ MF06036) expressed as CFU±SEM. Transfer efficiencies are calculated as the number of transfers per donor.
