# Supplementary material for: A Novel Biofilm Model System to Visualise Conjugal Transfer of Vancomycin Resistance by Environmental Enterococci
Source: Microorganisms. 2021 Apr 9;9(4):789. doi: 10.3390/microorganisms9040789 (PMC8070047; doi:10.3390/microorganisms9040789)
Supplement: Supplementary file 1 [file microorganisms-09-00789-s001.zip › Supplementary Figure S2.pdf]

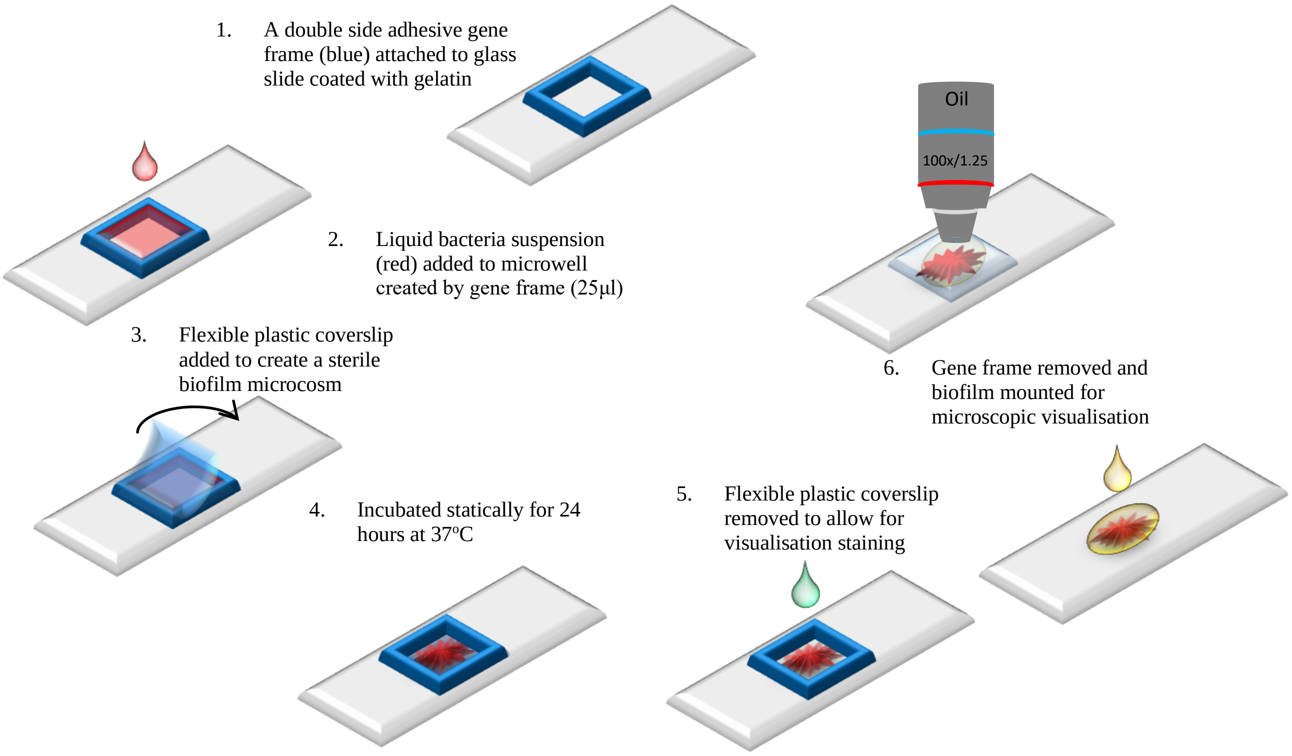

Supplemental Figure 2. The gene frame apparatus (GFA): A novel biofilm development tool for microscopic visualisation of fragile enterococcal biofilm.
